# Expatriate Family Adjustment: An Overview of Empirical Evidence on Challenges and Resources

**DOI:** 10.3389/fpsyg.2018.01207

**Published:** 2018-07-23

**Authors:** Mojca Filipič Sterle, Johnny R. J. Fontaine, Jan De Mol, Lesley L. Verhofstadt

**Affiliations:** ^1^Department of Experimental Clinical and Health Psychology, Ghent University, Ghent, Belgium; ^2^Department of Marital and Family Therapy, Faculty of Theology, University of Ljubljana, Ljubljana, Slovenia; ^3^Department of Personnel Management, Work and Organizational Psychology, Ghent University, Ghent, Belgium; ^4^Psychological Sciences Research Institute, Université catholique de Louvain, Louvain-la-Neuve, Belgium

**Keywords:** expatriate family adjustment, narrative review, challenges, resources, expatriates, family practice, adjustment disorders, third culture kids

## Abstract

The current theoretical paper presents a comprehensive overview of findings from research attempting to understand what happens with expatriates and their families while living abroad. Our paper draws on research on adjustment of individual family members (expatriates, their partners, and children) and families as a whole, across different literatures (e.g., cultural psychology, family psychology, stress literature). The key challenges of expatriation are discussed, as well as family members’ resources. Our findings lead to the following conclusions: First, there is lack of systematic research as studies are either missing a theoretical background or largely neglect the multi-informant approach. A comprehensive theory of expatriate family adjustment integrating multiple theoretical perspectives, including the culture identity formation and the impact of home country and host country culture, is called upon. Second, the majority of studies paid little attention to define the concept of family or failed to take into account the cultural aspect of relocation. Third, there is a call for more longitudinal studies including all family members as adjustment is a process that unfolds over time and therefore cannot be sufficiently explained by cross-sectional studies. Suggestions for future research and practical implications are provided, with a special focus on how families could be assisted during their adjustment process.

## Introduction

The vast research literature on expatriate adjustment has been long characterized by a predominant focus on individual adjustment of an *expatriate employee* (James et al., 2004). Despite some recent research on successful outcomes of expatriate family adjustment and growing awareness that expatriate families need to receive special attention before and during the assignment, challenges of international assignments are still generally underestimated, both by organizations and families (Lazarova et al., 2015). This is remarkable as family members’ inability to adjust to foreign environments has been noted as one of the most critical causes of expatriate *failure* (Fukuda and Chu, 1994; Haslberger and Brewster, 2008). Expatriate success has been the major focus of management perspective on expatriation, traditionally studying traditional corporate expatriates who were supported by the company. Stress and coping literature identified several stressors and hardships of expatriate life (Brown, 2008) and social capital theories tried to explain what kind of social support should be provided to expatriates in the host country (e.g., Copeland and Norell, 2002; Lauring and Selmer, 2010). Family systems theory was generally used as theoretical background to study adjustment of expatriate families and expatriate children (e.g., Van der Zee et al., 2007; Rosenbusch and Cseh, 2012). In contrast, cultural theories explaining the process of family adjustment to a new environment are lacking. The expatriate family adjustment literature needs a comprehensive up-to date general theory to incorporate different aspects of this very complex matter. The lack of an overview of findings resulting from different focuses taken in different domains of research on expatriate family adjustment provides a rationale for a narrative review of the research on this topic. More specifically, the aim of the current paper was to synthesize the contemporary research literature (family systems, family stress, cross-cultural adjustment, social support, identity theory, work-family literature) on expatriate family adjustment.

After conceptualizing the terms *expatriate*, *family* and *adjustment*, we outline the evidence on challenges and resources in the adjustment process of expatriates, partners, children, and an entire family system. Details will be provided about the major constructs studied, the methodology (designs of the studies), and the theoretical framework within which studies explored the expatriate experience of families. Major conclusions will be presented and implications for future research and practice will be discussed. We drew on empirical quantitative and qualitative studies published in English in peer-reviewed journals and listed in the Web of Science, Academic Search Complete and Google Scholar, in the last 30 years (between 1988 and 2018). In this paper we also refer to some theoretical articles and reviews, particularly when outlining definitions and discussing theoretical backgrounds of the reviewed studies. A narrative literature review as a type of a review article has been chosen because it allows the literature coverage and flexibility to deal with a wide range of issues (i.e., challenges and resources of expatriate family members) within a given comprehensive topic (i.e., expatriate family adjustment) (Collins and Fauser, 2005). The contributions of our narrative review consist of conclusions derived from a holistic interpretation of the current state of the literature on expatriate family adjustment and are based on the synthesis of the empirical studies that have focused on this topic.

## Conceptualization of Expatriate Family Adjustment

In the context of international work experience, acculturation is a dual process of cultural and psychological change that takes place as a result of contact between two or more cultural groups and their individual members and which involves various forms of mutual accommodation (Berry, 2005). The outcome of acculturation is a longer-term psychological and sociocultural *adjustment*, in other words, relatively stable changes that take place in an individual or a group in response to external demands (Berry, 2005). The acculturation literature identifies different types of global workers, such as sojourners, immigrants, refugees, expatriates, etc. (Sam and Berry, 2006). To clarify the distinction between different types of cultural groups, Berry et al. (2011) proposed the following criteria: (a) migration, (b) voluntariness, and (c) foreseen permanence. For *expatriates*, the profile includes migration, voluntariness, and no foreseen permanence. Moreover, expatriates further differentiate themselves on average by a high educational level, and if not self-initiated, by support from their organization.

Expatriates were further defined as individuals who move to another country, change a place of residence and have a specific goal to work in the new environment (Andresen et al., 2014); or as assignees across a range of assignment types involving international relocation (e.g., long-term, short-term, and extended business travel assignments) (McNulty, 2015). In the work-family literature, *family* is any combination of two life partners, with or without children (Caligiuri et al., 1998); or as two committed partners, where a partner refers to both spouses and significant others and it refers to a *traditional* expatriate situation where one partner works and one is unemployed (Lazarova et al., 2010). McNulty (2014, p. 5) provided the following comprehensive definition of *an* *expatriate family:* “married, de-facto, live-in, or long-term partners of the opposite or same sex, with or without children, with family members that reside in one or many locations; and legally separated or divorced (single) adults with children, with family members that reside in one or many locations.” This definition includes non-traditional types of expatriates which is a new field of enquiry evolving in recent research. It differs from traditional expatriates regarding their family composition (step, single parent, split, overseas adoption, multigenerational), family challenges (special needs or gifted children), family status (single expatriates, accompanying family members besides children), sexual orientation, and gender (McNulty and Hutchings, 2016).

Black and Stephens (1989) defined *adjustment* as a degree of fit or psychological comfort and familiarity that individuals feel with different aspects of the foreign culture. Shaffer and Harrison (2001) described personal adjustment as identity reformation where personal and social roles are redefined when attachment and routines established in one’s home countries are broken, thereby adding a link between culture and personality changes in the context of expatriate adjustment. Haslberger and Brewster (2009, p. 387) defined adjustment as follows: “Expatriates shall be called adjusted to a facet if they are effective in dealings in the new environment (in their own eyes and in the eyes of their hosts), perceive themselves as adequately knowledgeable about the local environment, and feel neutral or positive emotions overall.” Adjustment has been understood as a process that involves managing change, new experiences, and new challenges. As a positive outcome it can enrich expatriates’ lives (Kempen et al., 2015), however, failure to successfully deal with the challenges can result in mental health consequences (Brown, 2008). The underlying stressors are expatriate’s adjustment to a new job together with a move abroad, a partner giving up a job, children attending a new school, long periods of separation from their loved ones, occupying a new residence, changing family routines, a change in financial status, cultural differences, role conflict, etc. (Patterson, 1988; Hechanova et al., 2003; Haslberger and Brewster, 2008; Bahn, 2015). Some of the stressors caused by adapting to life in a new environment may remain unresolved and become ongoing tensions (i.e., strains) (Patterson, 1988), resulting in increased psychosocial distress (Silbiger and Pines, 2014), depression (Magdol, 2002), increased alcohol and substance abuse (Anderzén and Arnetz, 1997), decreased physical and mental health, lower marriage satisfaction and readiness to re-assign (Lazarova et al., 2015), and worsening subjective work environment (Anderzén and Arnetz, 1999). Their emotional complaints are linked to identity issues, uprooting, repeated goodbyes, losses, constant changes, and unresolved grief (Bushong, 2013).

Confrontation with stressors and challenges described above will trigger expatriates’ application of *resources* and coping behaviors (Patterson, 1988). Previous studies found several individual characteristics that modify stress response and foster the expatriate’s adjustment to a foreign environment, such as internal locus of control, self-esteem, education, good command of languages, past foreign experience, cultural intelligence, communication ability, extraversion, agreeableness, emotional stability, and open-mindedness (e.g., Caligiuri, 2000; Ali et al., 2003; Hechanova et al., 2003; Copeland, 2004; Holopainen and Björkman, 2005; Lin et al., 2012).

Not much empirical research, however, has focused on how *families* of expatriate workers–both individual members and family as a whole- deal with stress and challenges of expatriate assignments, and which resources impact their adjustment. This is surprising for multiple reasons: first, according to the 2016 Global mobility trends survey which included respondents from 163 global companies representing over 11 million employees, 73% of expatriates were accompanied by a partner and 52% of expatriates who accepted overseas assignment took their children with them (Brookfield Global Relocation Services, 2016). Second, family members’ inability to adjust to a foreign assignment has been identified as one of the most critical causes of expatriate failure (Haslberger and Brewster, 2008; Lazarova et al., 2010). Finally, it has been argued, that an expatriate assignment is often seen as offering to a family and its members an opportunity to enrich their cultural and general life (e.g., new international experiences, educational possibilities) (Suutari and Brewster, 2000; Richardson, 2006; Dickmann et al., 2008; Kempen et al., 2015).

Taken together, the literature on expatriate family adjustment shows that career decisions of expatriate workers are influenced by their family (and vice versa) and that understanding the challenges and the processes of adjustment of individual family members in determining the outcome of an expatriate family experience is therefore critical (McNulty and Selmer, 2017; Shockley et al., 2018).

In the following sections we will summarize the main empirical findings about the specific challenges and application of resources of expatriate workers’ trailing partners, children/adolescents, and families as a whole. In line with the aim of the current paper, the inclusion of studies in each section was based on their *unit of interest* (i.e., partners, children/adolescents and family as a whole). The *unit of measurement* in most studies was the individual. In the *partners* section, the informants were partners themselves or expatriate employees reporting about their partner; in the *children/adolescent* section – the informants were children reporting about themselves and expatriate employees/partners reporting about their children. In the *family* section informants were expatriates, partners and children. In other words, the measures were administered to individual informants, and they measured individuals’ perception of themselves and their families/relationships.

## Trailing Partner

### Crossover Effects

Within the HR framework, the most frequently reported reason for a failure in an international assignment (when defined as a premature return) was found an inability or an unwillingness of a partner to adapt to the foreign environment (Punnett, 1997; Haslberger and Brewster, 2008), together with a trailing partner’s career concerns (Lazarova et al., 2015). Similarly, Black and Stephens (1989; a cross-sectional study; 220 expatriate managers and 157 expatriate spouses; assigned in Asia), showed that partner’s positive opinion about the overseas assignment is predictive of their own adjustment, which is in its turn, highly correlated with the adjustment of expatriate managers.

Many studies have indeed shown *significant crossover effects* among partners (e.g., Black and Gregersen, 1991a,b; Forster, 1997). Van der Zee et al. (2005) conducted a cross-sectional empirical study in the Netherlands with a sample of expatriate partners from 21 home countries and found a crossover of stressors from the expatriate to their partner’s subjective well-being, and a crossover of the expatriates’ emotional distress to their partner’s distress and vice-versa. Based on the work-family and cross-cultural adjustment literature, Takeuchi et al. (2002) empirically tested and confirmed a crossover and spillover model of expatriate’s adjustment (cross-sectional study including 215 Japanese expatriates assigned in the midwestern United States, 169 spouses, and their superiors). Spillover effects related to the impact of expatriate attitudes in a particular domain (e.g., work) on other domains (e.g., home), whereas crossover effects related to the impact of expatriate attitudes on partner’s attitudes and vice versa. They found evidence for the reciprocal crossover effects between the cross-cultural adjustment of the expatriate worker and their partner. More specifically, a negative or a positive synergy between both partners had a significant impact on their cross-cultural adjustment (i.e., failure of one partner to adjust affected the other’s adjustment, causing a downward spiral of losses that could result in premature termination of the international assignment). Still in the framework of work-family interface, and integrating social capital and social networks theories, Lauring and Selmer (2010) conducted a systematic ethnographic field study using observation and semi-structured interviews with Danish expatriate partners in a compound in Saudi Arabia. They found that partners who feel well adjusted to the general environment in the host culture can have a positive influence on expatriates as they can support them with information on how to use transportation services, or in their social interaction, or even further the expatriates’ careers and repatriation opportunities by using different social strategies.

### Specific Challenges

Lack of preparation, relocation, and cultural novelty induce quite some stress for partners (Forster, 1997; a qualitative study with United Kingdom expatriate partners; Shaffer and Harrison, 2001). Some studies documented that expatriate partners have to link up more with the local culture as compared to the expatriate employee or their children (Ali et al., 2003; a study with 247 expatriate spouses from 29 different countries, the majority from the United Kingdom, the United States, Australia, and the Netherlands). Therefore, the adjustment challenges for partners are not only considered as different, but also greater (Punnett, 1997). According to a field study of 45 male expatriate accompanying partners in the Asia Pacific region conducted by Cole (2012), particularly male trailing partners feel isolated due to a small peer group; they clearly need assistance with establishing personal support network by joining a peer group in a host country. Partners often feel lost in a sense that they do not have an outside professional identity or a specific clarification of their family identity (Rosenbusch and Cseh, 2012). A lot of partners see their employment status change and lose their career because of a move which causes disturbance within home and lowers the interactional adjustment (i.e., interaction with the host–country nationals) (Shaffer and Harrison, 2001; Cole, 2011). In case where both partners pursue their careers in the host country, women seem to experience more work-personal life conflict than men (Mäkelä et al., 2017). Brown (2008) in a cross-sectional study in London, the United Kingdom, used a public sample of expatriate couples and found that dominant stressors of partners of expatriates were reduced self, local pressures, and isolation. More specifically, partners (as well as expatriates) reported to be stressed by spending insufficient time together, not having close friends to confide in, by concerns over children and family, and by feelings of uncertainty about their future after the current expatriate assignment. Similarly, an interesting study by Lazarova et al. (2015) highlighted the most common causes of expatriate failure were partner’s career concerns, partner’s resistance to move and marital breakdown. The latter has only recently been addressed in the research literature, although relationship issues appear to be a big challenge for expatriate couples which may result in expatriate divorce (McNulty, 2015). McNulty (2015) conducted a qualitative exploratory case-based study using data from 13 face-to-face interviews and 25 online survey participants. She found that expatriate marriages end in divorce because of two main reasons; either there has been a core issue in the marriage before expatriation (e.g., alcoholism), or one or both spouses are negatively influenced by expatriate culture to such an extent that it induces polarization behavior that is counter to how they would behave in their own culture (e.g., infidelity). In either case, findings showed that the outcomes of expatriate divorce were significant and may involve bankruptcy, homelessness, depression, alienation from children, even suicide. Taken together, the expatriate literature points to a more difficult situation of a trailing partner as compared to an expatriate employee (Cole, 2011). However, the literature also revealed some factors that may foster partners’ adjustment.

### Resources

The first category of resources consists of partners’ individual characteristics. Intercultural personality traits–emotional stability, social initiative, and open-mindedness- were found to be important resources for expatriate partners (and the expatriate employees’) psychological and sociocultural adjustment (Ali et al., 2003; Van Erp et al., 2014). Intercultural personality traits as coping resources for expatriate couples’ adjustment were explored by Van Erp et al. (2014), in a cross-sectional study with a sample of 98 Dutch expatriate couples (196 expatriates), and a longitudinal analysis of 45 couples from 43 different countries. They found the so-called compensation effect, whereby a partner’s lack of intercultural personality traits (as listed above) was compensated for by the other partner’s higher levels of those traits. High motivation, favorable opinion about the overseas assignment, previous expatriate experience, pre-move visit, cross cultural training and/or language training, host country language proficiency, social efficacy, self-efficacy and certainty about the duration of assignment proved to be positively related to partner’s adjustment (Black and Stephens, 1989; Shaffer and Harrison, 2001; Copeland, 2004).

The second category of resources includes identity reestablishment and feelings of psychological security. For example, drawing upon identity theory and the expatriate literature, Shaffer and Harrison (2001) studied spouse adjustment using a mixed method design with a sample of 211 expatriate couples in 37 countries and six continents. Findings showed that cross-cultural adjustment depends to some extent on whether partners can re-establish their identity in the new culture, including their individual/personal base of identity (i.e., language fluency), interpersonal/social base of identity (i.e., having preschool aged children), and environmental/situational base of identity (i.e., culture novelty and favorability of living conditions). Similar findings–on professional identity and social status- were reported by Copeland (2004). Herleman et al. (2008) found that a partner’s sense of comfort and psychological security in specific locations they regularly visit, a concept coming from Japanese culture called *Ibasho*, proved to be an important predictor of their adjustment and well-being. This study was conducted in Belgium and used a mixed method design with sample of 104 expatriate wives mainly coming from the United States, Canada, and the United Kingdom.

Thirdly, and at a more social level, company assistance prior and during expatriation, support from families, and support (e.g., network size, breadth of support, depth of support) from host country nationals, but also contacts with other expatriate partners, and time with old friends as well as new acquaintances were found to be essential to partners’ adjustment (De Cieri et al., 1991; Shaffer and Harrison, 2001; Caligiuri and Lazarova, 2002; Ali et al., 2003; Copeland, 2004). Copeland and Norell (2002) studied the role of social support within the framework of social support theory with 194 trailing partners (American women residing in 17 host countries in Europe, Asia, the Middle East, Latin America) and found that better adjusted women had participated in the decision to relocate, experienced fewer losses in friendships, had more functions of social support adequately met and could rely on the support from local rather than long-distance providers, and they were coming from families with higher cohesion. Further empirical evidence showed that family cohesion and adaptability (i.e., the ability to change and adapt to new environments while at the same time remaining closely tied to each other), open communication among partners (Ali et al., 2003), satisfaction with family relationships and extended family support (De Cieri et al., 1991; Shaffer and Harrison, 2001) facilitate partner’s adjustment. In a qualitative study Gupta et al. (2012) used the grounded theory methodology with 26 Indian trailing partners accompanying their partners on assignments in four continents (Asia, Europe, North America, and Australia). Findings of this study corroborated previous research such that the level of trailing partners’ adjustment was greatly impacted by cultural novelty, support from family, peers and the organization, and their personality. Moreover, they found that expatriates’ perceived gender-role ideology and marital obligations toward their partners played a significant role.

## Children and Adolescents

### Third Culture Kids (TCKs)

Pollock and Van Reken (2009) have introduced the following description of a TCK: *“A Third Culture Kid is a person who has spent a significant part of his or her development years outside the parent’s culture. The TCK builds relationships to all of the cultures, while not having full ownership in any. Although elements from each culture are assimilated into the TCK’s life experience, the sense of belonging is in relationship to others of similar background.”* (Pollock and Van Reken, 2009, p. 13). The identity formation of TCKs and their cultural and intellectual development is taking place in the third culture, particularly in the international environment in the host country (first culture is understood as parents’ culture and the second culture is a host culture). TCKs share more common experience to other TCKs than to their peers who grew up in their home or host cultures (Bonebright, 2010). Among difficulties, such as struggling with a sense of belonging and disruption of identity formation, having lived in different cultures also provided TCKs with skills to handle change, to be more open and accepting to different cultures and to successfully handle these differences. Bonebright (2010) in her review also pointed out the potential that adult TCKs can bring to HR looking for business expatriates. Besides being used to frequent travel and changes as part of an international mobile lifestyle and having good education and language skills, they also have experience of adjusting to a new work and life situation in a new location.

Selmer and Lam (2004) conducted a survey study with British expatriate adolescents (63 respondents living in Hong Kong, mean age 14 years), local Hong Kong adolescents ethnic Chinese (a sample of 103 adolescents, mean age 17 years), and a sample of British adolescents living in the United Kingdom with 88 respondents. They showed that British expatriate adolescents had distinct characteristics in terms of their perceptions of being international as well as their international mobility preferences and consequences. Moore and Barker (2012) were interested in cultural identity of third culture individuals and employed a biographical phenomenology or life story interviewing as a qualitative data collection method with a sample of 19 individuals between the ages of 18 and 44, of six nationalities, from 23 countries in all continents, with varied intercultural experiences. They found that TCKs possessed multiple identities or multicultural identity, they lacked clear sense of belonging but are competent intercultural communicators and perceive their experience as mainly beneficial.

### Crossover Effects

Expatriate’s work satisfaction has been found to positively affect children’s adjustment (Van der Zee et al., 2007). Further, the research has documented that effective adjustment of adolescents might lead an expatriate family to stay abroad longer than originally planned (Weeks et al., 2010). However, little is known about the extent that demands faced by children have on their parents’ adjustment. It has been noted that crossover effects of family stress to children need to be acknowledged and talked about within the family (Lazarova et al., 2015).

### Specific Challenges

Depending on their own age, children have to face additional challenges and these may have significant effects on the moving family as a whole. One of these challenges, described by Rosenbusch and Cseh (2012) is children’s confusion about their role (specifically gender role expectations), as a result of being raised in different cultures. Other challenges for young children are linked to loss of their home and their social network, change of schools, making of new friends, and learning a new language (Pollari and Bullock, 1988; McLachlan, 2008; Lazarova et al., 2015). Feelings of uncertainty, a sense of belonging to a culture and identity loss have been frequently reported (Ali, 2003; Moore and Barker, 2012; Rosenbusch and Cseh, 2012). Emotional instability and an ambivalent attachment style were identified to be important risk factors that made children more susceptible to adjustment problems (Ali, 2003; Van der Zee et al., 2007). In the framework of adolescent development theory and the concept of third culture kids, Weeks et al. (2010) used in-depth interviews to study the adjustment of expatriate 18 students age 14–19 of private international school in Shanghai, China, who were coming from the United States (the majority), Australia, Canada, Malaysia, and Philippines. They found that expatriate children have unique challenges of adjustment to a foreign environment, among which were the disruption of the identity formation process during their adolescence, concerns related to making friends, fitting in, and to be successful in school. One of the difficulties they tend to experience is that in their host culture they may stand out because of different look and usually they act differently than host country nationals. Lucier-Greer et al. (2015) explored normative and context risk factors and the role of relationships (family, informal networks, formal systems) as protective factors among adolescents from military families (a sample of 1036 adolescents between 11 and 18 years of age) located at four United States army installations, one of which was in Europe. They found that higher levels of cumulative risk experienced by adolescents were associated with more depressive symptoms, lower academic performance and lower persistence (Lucier-Greer et al., 2015). International move can disrupt adolescents’ identity formation process, which is characterized by a growth toward more autonomy, becoming more independent from parents, and peers becoming new attachment figures.

### Resources

At the individual level, being open-minded (i.e., understanding that cultures are different and that people around the world have different perspectives on a variety of issues) was reported by adolescents to be key to adjusting well (Weeks et al., 2010). Secure attachment, emotional stability, and high level of social initiative were found to foster children’s adjustment (Ali, 2003; Van der Zee et al., 2007). Because of having multiple experiences with different situations and people it is easier for them to interact with different people and to adapt to new situations (Moore and Barker, 2012).

In terms of family resources, Van der Zee et al. (2007) studied family characteristics such as family adaptability (i.e., the extent to which a family is flexible and able to change its functioning; Olson et al., 1984), family cohesion (i.e., the amount of emotional bonding between family members; Olson et al., 1984), and family communication (i.e., the tool through which families can create a shared sense of meaning, develop and orchestrate coping strategies, and maintain harmony and balance; McCubbin et al., 1996). To examine the determinants of effective coping with cultural transition, they used a survey with a sample of 104 expatriate children and adolescents from 21 different home countries (the majority from the Netherlands, Germany, Switzerland, Belgium; who lived in 37 different countries; the majority in the Netherlands, Singapore, and France). They found that all three characteristics contributed to higher levels of intercultural adjustment of children, with family cohesion being the strongest predictor of both quality of life and sociocultural adjustment of expatriate children and adolescents. Traits and attachment styles were directly associated with better adjustment, and moreover, they also moderated the relationship between family and work-related factors and intercultural adjustment.

Family cohesion may also impact expatriate children’s ability to establish and maintain friendships with other children in the host country (Caligiuri et al., 1998). In the early stage of a relocation to an unfamiliar environment, family members need to rely primarily on each other. The emotional support from parents and siblings and good discussion with parents about the move, where parents show sensitivity to children’s specific needs in the host country, were found as important facilitators in the adjustment process of children and teenagers (De Leon and McPartlin, 1995; Ali, 2003; Weeks et al., 2010; Lazarova et al., 2015).

Another important social resource for children and adolescents is the support they receive from friends, primarily at school (Weeks et al., 2010). Teenagers don’t seem to be bothered by the fact that they are often isolated from the host culture (Weeks et al., 2010), however, they really seem to need friendships with peers who speak their mother tongue. Overall, some evidence shows that family support and informal networks buffer against depressive symptoms with adolescents and their academic performance as well as persistence were higher (Lucier-Greer et al., 2015).

## Family as a Whole

Expatriation demands major changes in family roles and living circumstances. Takeuchi (2010) and Lämsä et al. (2017) underscored the importance of considering the family and its members as stakeholders of a company to examine family’s expectations with regard to company support. Our overview of empirical evidence of the research on expatriate family adjustment showed that there is a limited number of studies that explored expatriate family as a unit and included all family members as informants. Below we discuss the studies that examined family level variables or explicitly focused on family adjustment (see e.g., Caligiuri et al., 1998; McLachlan, 2008; Rosenbusch and Cseh, 2012; Lazarova et al., 2015). Lazarova et al. (2015) conducted a large study using a convenience sampling approach with 656 expatriates, expatriate spouses and teenage children coming from 51 home countries and assigned in 77 countries using work-life balance, family systems, and crossover theory to explore family narratives on international mobility. Findings showed that a successful movable family should be adventurous, have a sense of humor and good communication where all members ‘pull in the same direction’ and all members are treated as important in family decisions, family members need to make an effort to socialize outside of the family and all the members should be committed to the move. Family members may have different needs that also surface at different times, and some tensions linked to the strains of moving, nevertheless, these stressful events may also bring family together. On the other hand, there is the need to perform, to be brave and to keep going, although at times it is barely manageable. Further, this study pointed to the changing face of expatriate family including both parents and children.

Indeed, there is a growing body of research on non-traditional family forms, such as women as breadwinners, single parents, step families, same sex families with dual careers and children (McNulty and Selmer, 2017). McNulty (2014) reported on a case study with a sample of four female western expatriates living in Singapore, China, Brussels, and North Carolina – a single parent, overseas adoption, split family and lesbian assignees in their breadwinner roles. Fischlmayr and Puchmüller (2016) used social capital theory as a theoretical base for their study on the experiences of 25 female international business travelers living in dual-career families from four Western and non-Western countries on four different continents. The analysis of the interviews showed both similar and different experiences (i.e., childcare and support networks, and social acceptance), and understanding of integrating family and career life as female non-traditional expatriates across cultures.

An expatriate assignment offers opportunities for families: relocating may bring the family closer, especially if the host country is marked by limited social resources and strong cultural differences (Copeland and Norell, 2002). De Cieri et al. (1991) found that a large proportion of women commented that their relationships with their children had become closer through the relocation, because they had similar challenges. It was documented that the expatriate experience usually starts with great excitement and positive expectations (Punnett, 1997; Osland, 2000). In an interesting qualitative study by Osland (2000), expatriates reported that the stage of leaving home and crossing the physical and cultural threshold of a foreign land lasts about 6 months and is characterized by strangeness, difficulties, ups and downs, by the feelings of uncertainty (questioning their own identity, their values, and their understanding of everyday life), a sense of uneasy responsibility for uprooting their family with no guarantee that every family member will adjust to the new culture, and by intense, accelerated learning. After their return home expatriates reported being proud of succeeding difficult work challenges, making it ‘on their own,’ feeling ‘special,’ and taking pride in their ability to acculturate and adapt to change.

### Spillover Effects

Caligiuri et al. (1998) were the first to report on spillover effects between family life and work adjustment: if expatriates are well adjusted to working in the host country, their positive feelings will spill over to their family and facilitate family’s cross-cultural adjustment. This study used family systems theory as theoretical background and collected data from 110 families (mostly coming from Canada, the United States, and the United Kingdom) on global assignments in 26 different countries. Some other studies confirmed a positive spillover of adjustment to expatriates’ partners and children (Ali, 2003; Trompetter et al., 2016). Van der Zee et al. (2005) reported a negative spillover of expatriates’ home demands to their work roles. Shaffer and Harrison (1998) showed that expatriates with greater family responsibilities paid increasingly more attention to non-work factors in making their withdrawal decisions.

### Specific Challenges

Rosenbusch and Cseh (2012) used family systems theory and expatriate adjustment as theoretical knowledge base to study cross-cultural adjustment of expatriate families in a multinational organization based in the United States. They recruited a sample of 15 expatriate families (111 expatriates, 15 spouses, and 7 adolescent children) and applied a case study with mixed method approach. Cultural, relational and psychological stressors had the highest impact on the cross-cultural adjustment, among which cultural stress seemed to be the greatest. Overcoming cultural differences, grasping the art of a new language and being understood by host country nationals were found big challenges in the adjustment process of expatriate families. Challenging were also physical health (i.e., weight gain), physical stress, feelings of loneliness, struggle to maintain a sense of stability and comfort within the family unit, attempts to make new friends and to keep in touch with old ones (Rosenbusch and Cseh, 2012). Emotional distress (i.e., anxiety or depression) may result from expatriate family’s unsuccessful attempts of adjustment (Rosenbusch and Cseh, 2012). A relocation takes extra toll on marriage and it has been argued that expatriate divorce increases stress and psychological trauma as it involves separation and custody disputes across geographical boundaries (McNulty, 2015). Extreme novelty, stress of a new environment, and expatriate’s lack of knowledge about how to obtain social reinforcement in the new culture, often compels expatriate families to seek professional help (Osland, 2000) and family counseling (Lazarova et al., 2015). It has been noted that special attention needs to be given to non-traditional family structures, such as status reversal marriage (i.e., females as breadwinners), single parents, split families and gay partnerships (McNulty, 2014).

### Resources

A few studies focused on *family characteristics/dynamics* that may foster or inhibit adjustment of its individual members or the family as a whole. Having a sense of adventure, good and open communication, commitment to the move of all family members, trying to socialize outside the family unit were all found to facilitate family adjustment (Lazarova et al., 2015). Rosenbusch and Cseh (2012) showed that of the six components of family flexibility, the components of roles, rules, leadership and assertiveness had the most impact on cross-cultural adjustment of expatriate families. More specifically, families experienced lack of *role* differentiation and were in need of specific clarification of family identity. Partners reported feelings of loss outside the professional identity due to career interruption. *Leadership* within the family seemed to be a core issue during the move, as family members found that part of their responsibility was to assist other family members in adjusting to a new environment. Sharing their opinions with one another (*assertiveness*) and staying connected as a family was important for family members. Families with a supportive climate, good family communication, and a positive perception of the international assignment experienced more successful adjustment (Caligiuri et al., 1998; Copeland and Norell, 2002). Also, family members’ *satisfaction with their family relationships* has been shown to be significantly associated with psychological adjustment to relocation and satisfaction with life throughout the expatriation (De Cieri et al., 1991; Richardson, 2006 – informants were expatriates). In particular, healthy relationships between partners were found critical for a successful expatriate family (Lazarova et al., 2015).

The second category of family resources are *external to the family*. Help from the company in dealing with financial concerns related to the move and life in a new country, and good organizational and practical support, including providing contacts in the new country are all important support systems for adjustment of an expatriate family (Lazarova et al., 2015). Active involvement within a church, school, youth organization, employing organization, health or welfare organization in the host country helps family members to adjust quicker to a new location (Cornille, 1993). Schools (most often international ones) can offer support for families by encouraging dialog among families and the school and facilitate parent-adolescent communication during relocation (McLachlan, 2008 – a qualitative study with 45 families at an international school in southern England).

A long-distance family support is crucial during assignment-imposed separation (Richardson, 2006; Starr and Currie, 2009 – both empirical studies drew from expatriates as informants), and different forms of electronic communication allow family members to stay connected with their extended family and friends (Rosenbusch and Cseh, 2012). The internet and social media is increasingly providing a new form of social communication with family and friends and thus enable emotional support provision from them (Haslberger and Brewster, 2008), and which were also found to be reasonably good predictors of levels of perceived social support, loneliness and depressive states of adult expatriates shortly after their residential move (Shklovski et al., 2006).

## Conclusion and Implications

### Summary of Findings

Based on the empirical evidence from the studies included in our review, the following conclusions can be made about the *challenges* and *stressors* that come along with living as an expatriate family. Cultural novelty, lack of preparation and relocation (financial) support, loss of home, change of social environment, increased demands related to organizing life in a new location (i.e., schooling system, learning about local culture and language, daily hassles, new work situation for expatriate employees), adjustment to work (expatriate employee), together with feelings of uncertainty, up-rooting and isolation are stressors that all family members need to face to certain extent (e.g., Osland, 2000; Haslberger and Brewster, 2008; Rosenbusch and Cseh, 2012; Lazarova et al., 2015).

However, there are some differences among family members, too. Children and adolescents are most concerned by fitting into new schools and making new friends and not so much by learning the local language and creating social networks outside school (e.g., Weeks et al., 2010). Trailing partners, on the other hand, are preoccupied with finding ways to organize family life, learning the culture and language of the host country, finding a job, and can feel isolated and lost without outside professional identity (e.g., Brown, 2008; Cole, 2011; Rosenbusch and Cseh, 2012; Lazarova et al., 2015). Establishing social contacts with local nationals and other expatriates, getting familiar with local culture and languages are necessary and important for the whole family (Black and Stephens, 1989). International experience can bring family members together, which is an important positive outcome of expatriation, however, family as a whole may also feel isolated and lonely (e.g., De Cieri et al., 1991; Rosenbusch and Cseh, 2012). Taken together, during their adjustment process, expatriate families are confronted with the following challenges: children’s education, partners’ resistance and career issues, location difficulties, cultural adjustment, language issues, and support for other family members.

Our narrative review also documents the process by which individuals and families cope with the challenges and stressors described above (i.e., their *coping resources*). Personal/psychological resources such as open-mindedness, emotional stability, high level of social initiative (e.g., Ali et al., 2003; Weeks et al., 2010; Van Erp et al., 2014) together with family resources such as flexibility, adaptability, and cohesion (e.g., Caligiuri, 2000; Ali et al., 2003; Van der Zee et al., 2007) act as resources for expatriates as well as for their family members. Good relationships within the family and beyond contribute to the subjective well-being of expatriates and their family members (e.g., Richardson, 2006; Lazarova et al., 2015). Turning to more social-level resources, maintaining contact with the extended family (Richardson, 2006), friends and former colleagues–with the use of social media and internet- helps family members to overcome feelings of loneliness and isolation (e.g., Shklovski et al., 2006; Rosenbusch and Cseh, 2012). Talking to other people when in need of emotional support and asking for help with the everyday engagements alleviates distress with expatriates (Caligiuri and Lazarova, 2002). Social support networks play an important role in the adjustment process – although expatriates, partners and children may use different ways to integrate socially. For children, good integration at their school is crucial (Weeks et al., 2010), for partners support from host country nationals (e.g., Shaffer and Harrison, 2001; Copeland and Norell, 2002), and for expatriates and partners organizational support and company assistance are important (e.g., Ali et al., 2003; Cole, 2011, 2012; Lazarova et al., 2015).

The third conclusion concerns *reciprocal influence* between family members in terms of stressors, application of resources, and adjustment. Crossover effects (for instance of stressors, subjective well-being, emotional distress) between partners have been documented in the literature (e.g., Takeuchi et al., 2002; Van der Zee et al., 2005; Lauring and Selmer, 2010). Also, family situation and work adjustment of expatriate employees are strongly related (Caligiuri et al., 1998). Finally, crossover effects for all family members, including children, need to be taken into account when relocating with children (Lazarova et al., 2015).

The fourth conclusion concerns the *methodological characteristics* of the studies included in our review. At the level of study designs, we can conclude that there is a growing body of qualitative studies attempting to provide insights into the subjective experience of expatriate family members, or studies using both quantitative and qualitative methods (see e.g., Lauring and Selmer, 2010; Lazarova et al., 2015; McNulty, 2015; Fischlmayr and Puchmüller, 2016). Qualitative studies mostly used interviews to gather data from expatriates to understand their expatriate complexity. The research on expatriate families, expatriate children and TCKs, is still evolving and such qualitative designs are helpful for better understanding the lived experience of the emerging expatriate (sub)groups. While most studies used methodological perspectives of cross-cultural psychology, cultural psychology perspectives are barely presented in the area of expatriate family adjustment. Research including empirical ethnographic field studies that incorporate the lived experience of a host country culture is scarce (see e.g., Lauring and Selmer, 2010 as an important exception). Finally, the majority of quantitative studies used cross-sectional designs, and longitudinal study designs are hardly applied.

Concerning the *samples and geographical location* we can conclude that the majority of studies used samples with English speaking expatriates, mainly coming from the United States, Canada, the United Kingdom, and Australia. The exceptions used samples from Asia (e.g., Japan, India), and Europe (mostly from Western Europe). There is a huge gap in studies featuring populations from Central, Southern and Eastern Europe, Africa, Latin America and most of Asia, and studies using non-English speaking samples.

Fifth, at the level of the *theoretical background*, it can be concluded that family systems theories, cross-cultural adjustment, expatriate literature and social support network theory prevail as the knowledge base for the research. Another observation is that management theories have studied adjustment through the lens of success – for a company and also for the expatriate and expatriate family. The successful assignment presents less costs for the organization. Cultural perspectives, on the other hand, remain largely unaddressed (see Shaffer and Harrison, 2001; Rosenbusch and Cseh, 2012, for notable exceptions).

Sixth, and also at the conceptual level is the observation that the majority of studies failed to explain the *definition of a family* used in the study (see Caligiuri et al., 1998; Lazarova et al., 2010; McNulty, 2014, for exceptions). It is understood that they involve parents and children, however, the traditional family definition is no longer useful because of the changing family constellations. The most comprehensive family definition was proposed by McNulty (2014) who also included non-traditional family forms such as long-term partners of opposite sex, single adults with children, and families of which members may reside in different locations. There is a huge gap in the research about self-initiated expatriate families. The majority of studies used the term *spouse* or *wife* to refer to a partner accompanying (usually) male expatriates on assignment. For our review we therefore decided to use the term *trailing partner* to refer to a significant person in an expatriate life that accompanies them on international assignment.

Taken together, the majority of the empirical research used quantitative methods studying expatriates in a given context, the focus in the existing research is predominately on challenges and hardships of expatriate life whereby the positive experiences of expatriation have been largely neglected. During the last decade the research agendas are also shifting from company based western male expatriates to new forms of expatriation and new types of non-traditional families.

### Directions for Future Research

Based on the findings of our review, we can conclude that despite the fact that research on expatriate family adjustment is growing (Caligiuri et al., 1998; Rosenbusch and Cseh, 2012; McNulty, 2014; Lazarova et al., 2015), the available empirical evidence is limited. Our review identifies the following *avenues for future research*.

First, the neglected area remains *adjustment of expatriate children and adolescents*, which cannot be explained by current adult-focused theories as children have different challenges than their parents (Shaffer and Harrison, 2001). The existent TCK literature tends to rely upon the work of Pollock and Van Reken (2009). More research is needed about identity formation and challenges that come along with living abroad, and particularly about resources that they can apply while growing up in the international environment. Namely, their culturally mobile upbringing defines them as being the very essence of multicultural individuals in a global society (Moore and Barker, 2012). With many new forms of non-traditional expatriate families, TCKs’ profiles are also changing into even more globally oriented and independent. This is very important as TCKs have a great potential to become future expatriates and can be in advantage in the world labor market due to their specific international experiences (Bonebright, 2010). The literature on TCKs needs an all-inclusive definition to explain the specifics of the identity formation of TCKs, taking into account the characteristics of different cultures and also the influence of parents’ culture. Furthermore, different types of families and also multicultural families need to be considered when studying the international experience of young people.

Second, more research is needed on the *reciprocal influence* between all family members (e.g., impact of expatriates on partners; impact of children on parents and vice versa). Since families living in a foreign country often become closer and need to rely on their own resources (De Cieri et al., 1991; Copeland and Norell, 2002), their role to support each other to overcome potential crises may be even more important than in their home country (in which community/social sources of support are more available).

Third, so far studies on expatriate adjustment have mostly been overly *restrictive* in their focus and only a limited number of variables were investigated (Takeuchi, 2010). Therefore, future research should broaden its scope to different stress variables (e.g., chronic strains, daily hassles) as well as to different outcome variables (e.g., short term crisis, long term adjustment). Further research should include the adaptation to changing family roles, to map relationships among forms of adjustment and to offer a systematic way to group adjustment antecedents (Lazarova et al., 2010). Recently published articles on expatriate family experience (e.g., Lazarova et al., 2015; McNulty, 2015) call for more research on topics that do not focus on expatriate success but rather give in-depth insight into experience of expatriation for a family. Additionally, with the increased globalization, studies on expatriation could learn more from migration studies to improve conceptual refinements of concepts of expatriation and to deepen the knowledge base and provide relevant practical advice for different types of expatriates (Andresen et al., 2014).

Fourth, many studies examining expatriate family adjustment lack a *theoretical background* or invoke the stressor-stress-strain perspective (Bhaskar-Shrinivas et al., 2005), or the work-family literature as their underlying theoretical basis (see Caligiuri et al., 1998; Van der Zee et al., 2007; Takeuchi, 2010; Rosenbusch and Cseh, 2012; Lazarova et al., 2015, as important exceptions). Studies on expatriate families, however, could integrate family psychology literature, family systems theory, and family stress models, positive psychology, and in particular, cultural psychology and cultural identity formation. A different culture and language barriers in the host country may be a challenging experience for expatriates, their partners and children involving the need to restructure, develop, and adapt in response to the requirements of the new environment. Capturing the cultural experience of the expatriate family would add to the existing knowledge where currently family and its members seem to be the sole generators of their adjustment process. More research interest is needed to better understand the interface between expatriate family adjustment and cultural aspects of relocation, and specifically, into the identity change of expatriate family members and family as a whole.

Fifth, on the *methodological* level, there is a need for longitudinal designs that examine adjustment as a long-term process rather than a momentary event (Haslberger and Brewster, 2009). Most of the studies included in the current review were cross-sectional in nature and cannot inform us about the directionality of potential causal relationships between the variables under study. Qualitative designs including ethnographic field studies in different parts of the world and different cultures will be particularly useful to further our understanding of family members’ perception of their experience and meaning making during international assignments. So far cultural psychologists have not taken much interest in the research field. However, the understanding of expatriate family adjustment could be largely enriched and refined by cultural psychology’s specific concept of culture, its interest in thick descriptions and its preference for qualitative/hermeneutic approaches. More qualitative studies are needed also to provide insights and understanding of expatriate experience, particularly for non-traditional family compositions. For example, qualitative method interpretative phenomenological analysis (Smith et al., 2009) could provide insights into the expatriates’ subjective lived experience as this method is suitable to gain rich understandings of topics with little theoretical and empirical evidence. Further, quantitative studies should include large samples of homogenous groups of expatriates and aim to explain different constructs and processes related to expatriate family adjustment. More mixed methods research designs are called for to gain more knowledge on the breadth and depth of the expatriate family experience of adjustment. Replication of findings with larger and more diverse samples (e.g., across countries of location of assignments) is also needed (Herleman et al., 2008). Particularly, scholars should try to study different cultures in different parts of the world, as opposed to using mostly English-speaking samples from western countries.

Sixth, our recommendation points to the necessity of studies using a *multi-informant approach* where all family members – expatriates, trailing partners and children/adolescents – report on the variables of interest. The impression from the existing research is that such approach with large samples of expatriate families is difficult to apply (Takeuchi, 2010). One plausible explanation could be that expatriate families are probably very difficult to recruit for research because of increased stress and lack of time after the move. Also, there is no particular spot where they report to when they come to live in a host country. Therefore, more research effort and perhaps collaboration with scholars in different countries and cultures should be enhanced to produce studies across different cultures.

Seventh, many studies have looked into the relationship between *personality* and cross-cultural adjustment, however, very few focused on partners’ and children’s personality traits (see Ali et al., 2003; Van der Zee et al., 2007, for exceptions). Furthermore, we need more studies to focus on the positive side of expatriation for a family and how to address motives for international life.

Eighth, there is a call for more research on *new family forms* and non-traditional family structures, blended families with step-parents and half-siblings from prior relationships, single parents and status reversal marriages (McNulty, 2014), the emerging self-initiated expatriate families, and dual-careers families.

Ninth, as research points to the fact that an expatriate assignment may affect the psychological well-being of the family system as a whole as well as each individual family member, attention should be given to the development of *clinical interventions* with the expatriate population. Feelings of alienation, uprooting, constant changes and goodbyes are common complaints expatriates which expatriates can address in psychological counseling (Bushong, 2013). Specifics of multicultural counseling combined with family therapy could be useful professional support for families during their adjustment process. Findings of this narrative review therefore point to the need for future research on relational and family processes (i.e., dynamics, interactions, and stories) that influence the decision to move back or to prolong the stay.

Finally, there is a call for *more research* and more publishing on expatriate family adjustment. As mentioned above, expatriate families may be a difficult sample to recruit for the research. Further, one might argue that there is more research on expatriate families actually conducted than it appears in peer-reviewed journals.

### Practical Implications

Based on our narrative review on expatriate family adjustment, some practical and clinical implications can be outlined. For example, families could benefit from pre-departure cross-cultural and language training (Punnett, 1997; Copeland, 2004). During this training, the specifics of the host culture, past foreign expatriate experience, language skills, intercultural competences, and personal resources of the whole family could be targeted (Shaffer et al., 2006; Van Erp et al., 2014). The preparation part should also not overlook the importance of family members’ *perception* of and *motives* for the international relocation (Suutari and Brewster, 2000; Dickmann et al., 2008). Companies sending families on international assignments should be encouraged to include all family members in the pre-departure training (Shaffer and Harrison, 2001) where their different roles and expectations should be taken into account. Family counseling could forewarn of the upcoming changes and clarify family roles and family functioning, and could alleviate problems (Lazarova et al., 2010; Rosenbusch and Cseh, 2012). Additionally, more emphasis should be put on explaining the motives and positive aspects of relocation.

The preparation before the move and the actual process of adjustment may be highly influenced by the nature of the host culture. Particularly, it should be acknowledged that there is a difference if the host country is multicultural with different sub-cultures (e.g., the United States, big cities, such as London, Brussels, etc.) or monocultural (e.g., Japan). In cases where expatriate parents belong to one (the same) culture, they may not be completely aware that their children growing up as TCKs have different challenges. Therefore, it is of huge importance that parents receive counseling about how to support children during their most crucial developmental years, taking into account their identity formation and their developmental needs. While parents may be struggling with homesickness and planning their eventual return to their home country, for children the move may provoke additional stress as they may perceive it as adjusting to a new culture. TCKs belong to a ‘third culture’ which is placeless, and their restlessness and feeling uprooted may lead them to change places over and over again. TCKs feel best among other people with similar experiences which parents may find hard to understand and accept. In short, TCKs are different from their parents in terms of their cultural identity and families need to be educated and supported to deal with this challenge.

The possibility and availability of psychological support (e.g., family counseling) in the new location should be discussed with the family. Partners could specifically focus on how to use their time and resources when abroad (Lauring and Selmer, 2010). Direct communication and support between the company and trailing partner could facilitate adjustment of the whole family, as it is usually trailing partners who have to deal more with hassles of relocation (Lazarova et al., 2015). Children and teenagers could be prepared for the international assignment through video information about the life in the new school and friendships abroad (Weeks et al., 2010). Further, family members who are moving abroad and host country nationals should be put in contact before the departure so that hosts in the host countries could play an active role in the preparation activities.

Even with the most thorough pre-departure training families cannot avoid experiencing some degree of adjustment stress shortly after the relocation, and therefore some follow-up on the adjustment process after the move is warranted. For example, host country nationals could be considered to assist newcomer expatriate families with learning about the host culture and local customs in the new location (Osland, 2000). In particular human resources management could add value by providing adjustment assistance within the expatriate communities. For example, by supporting the development of friendships in the new environment (i.e., community groups, workplaces and online social media) (Bahn, 2015). Furthermore, employer provided career assistance and consideration of roles and responsibilities of both partners is needed for expatriate partners who plan to continue their career in the host country (Cole, 2011; Lazarova et al., 2015; Mäkelä et al., 2017). To be able to offer clear guidelines on how children facing many relocations in their life can obtain some degree of sense of stability when their family moves on international assignments, more research is needed on the nature of adjustment of children and teenagers.

In sum, our narrative review provides a summary of contemporary findings on expatriate family adjustment, including identification of challenges as well as personal, family, and community resources that foster adjustment of family members. Notably, clear conceptualization of expatriate family or expatriate family adjustment is needed. A general theory of expatriate family adjustment is called upon that would in a comprehensive way integrate multiple theoretical perspectives on expatriate family adjustment; work-family literature, adjustment and expatriate literature, stress and positive psychology, cultural and cross-cultural psychology, social theories, work transitions, family functioning, family relations, different types of families, and communication. Further, studies should not neglect culture identity formation of children and the impact of both home country and host country cultures. In particular, research using cultural psychology perspective is needed to enrich the understanding of expatriate family experience. Finally, more research should focus on shedding light on positive outcomes and opportunities of expatriate families.

Our narrative review represents an important contribution to expatriate family adjustment literature. It may serve as an important source of knowledge for experts in the field of expatriate family adjustment and related fields of research, such as cultural, cross-cultural psychology, family and organizational psychology. Because of its broad scope it can be accessible to broader audience, such as HR experts, teachers in international schools, clinicians working with expatriates, and of course present and future expatriate families.

## Author Contributions

MFS and JF conceived the contents of the review. MFS reviewed the papers and drafted the manuscript. LV conceived the structure of the article. LV, JF, and JDM edited the whole manuscript. All authors read and approved the final manuscript.

## Conflict of Interest Statement

The authors declare that the research was conducted in the absence of any commercial or financial relationships that could be construed as a potential conflict of interest.
